# Development of an Oral Epithelial Ex Vivo Organ Culture Model for Biocompatibility and Permeability Assessment of Biomaterials

**DOI:** 10.3390/bioengineering11101035

**Published:** 2024-10-17

**Authors:** Foteini Machla, Chrysanthi Bekiari, Paraskevi Kyriaki Monou, Evangelia Kofidou, Astero Maria Theodosaki, Orestis L. Katsamenis, Vasileios Zisis, Maria Kokoti, Athina Bakopoulou, Dimitrios Fatouros, Dimitrios Andreadis

**Affiliations:** 1 Department of Prosthodontics, Dental and Craniofacial Bioengineering and Applied Biomaterials, School of Dentistry, Faculty of Health Sciences, Aristotle University of Thessaloniki, 54124 Thessaloniki, Greeceatheodoe@dent.auth.gr (A.M.T.);; 2 Laboratory of Anatomy and Histology, Veterinary School, Aristotle University of Thessaloniki, 54124 Thessaloniki, Greece; chmpekia@vet.auth.gr (C.B.); evikofidou@gmail.com (E.K.); 3 Department of Pharmaceutical Technology, School of Pharmacy, Faculty of Health Sciences, Aristotle University of Thessaloniki, 54124 Thessaloniki, Greece; paraskemd@pharm.auth.gr (P.K.M.); dfatouro@pharm.auth.gr (D.F.); 4 Center for Interdisciplinary Research and Innovation (CIRI-AUTH), 57001 Thessaloniki, Greece; 5 μ-VIS X-ray Imaging Centre, Faculty of Engineering and the Environment, University of Southampton, Southampton SO17 1BJ, UK; 6 Institute for Life Sciences, University of Southampton, Southampton SO17 1BJ, UK; 7 Department of Oral Medicine/Pathology, School of Dentistry, Faculty of Health Sciences, Aristotle University of Thessaloniki, 54124 Thessaloniki, Greece

**Keywords:** organ/tissue culture model, oral mucosa ex vivo analog, epithelial barrier, permeability device

## Abstract

In the present study, a customized device (Epi-ExPer) was designed and fabricated to facilitate an epithelial organ culture, allowing for controlled exposure to exogenous chemical stimuli and accommodating the evaluation of permeation of the tissue after treatment. The Epi-ExPer system was fabricated using a stereolithography (SLA)-based additive manufacturing (AM) method. Human and porcine oral epithelial mucosa tissues were inserted into the device and exposed to resinous monomers commonly released by dental restorative materials. The effect of these xenobiotics on the morphology, viability, permeability, and expression of relevant markers of the oral epithelium was evaluated. Tissue culture could be performed with the desired orientation of air-liquid interface (ALI) conditions, and exposure to xenobiotics was undertaken in a spatially guarded and reproducible manner. Among the selected monomers, HEMA and TEGDMA reduced tissue viability at high concentrations, while tissue permeability was increased by the latter. Xenobiotics affected the histological image by introducing the vacuolar degeneration of epithelial cells and increasing the expression of panCytokeratin (pCK). Epi-ExPer device offers a simple, precise, and reproducible study system to evaluate interactions of oral mucosa with external stimuli, providing a biocompatibility and permeability assessment tool aiming to an enhanced in vitro/ex vivo-to-in vivo extrapolation (IVIVE) that complies with European Union (EU) and Food and Durg Administration (FDI) policies.

## 1. Introduction

The oral mucosa, the mucous membrane lining the mouth cavity, consists of a complex multilayered tissue with a protective barrier function, immune-related activity, and specialized functions, such as the production and secretion of saliva. This mucosa comprises a superficial multilayered epithelium, which can be keratinized, parakeratinized, or non-keratinized, depending on the location in the oral cavity, and is separated from the subepithelial compartment by the basement membrane. The subepithelial compartment is composed of various cell types and tissue structures, including adipose, connective, vascular, neural, osseous, and muscular tissues, as well as spherical structures the minor salivary glands, which are responsible for saliva secretion [1]. Microorganisms, food and environmental components, chemicals, drugs, and dental materials, such as metals and xenobiotics released by resin-based restorative materials, come into contact with the oral epithelium and can permeate it, reaching the subepithelial layers and dispersing systemically via the blood vessels. Meanwhile, parameters, such as the temperature, time, saliva pH, and mechanical forces through mastication or chemical influence, contribute to a multifactorial environment, further triggering the release of xenobiotics from dental materials into the oral cavity and leading to significant impacts on local and systemic health and disease [2,3,4].

Taking into consideration the paramount barrier function of the oral mucosa and the continuous development of novel dental materials, as well as drugs with an oral transmucosal administration delivery pathway, an unmet need has risen during the past years to develop reliable biocompatibility assessment systems that recapitulate the structure and barrier function of the oral mucosa [5]. These systems aim to serve as alternatives to animal testing in compliance with EU directives requiring the replacement, reduction, and refinement of laboratory animal use in research and development (3Rs rule) [6], as well as the Food and Drug Administration (FDA) rendering testing on animals optional [7]. While several efforts have been made to develop 3D oral mucosa analogs using tissue engineering approaches, such as epithelial barriers [8,9,10,11], full-thickness gingival equivalents [10,12,13,14,15,16], and biofabricated full-thickness gingiva-on-chips [17], these models, although advantageous compared to conventional 2D culture systems, still fall short of fully recapitulating the complex structure and function of the oral mucosa [18,19]. Therefore, the greatest challenge is to develop ex vivo systems with an enhanced in vitro-to-in vivo (IVIVE) extrapolation ratio.

**Figure 1:**
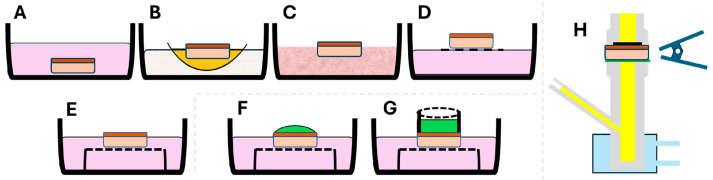
Schematic representation of the existing organ/tissue culture models (**A**–**E**), exposure methods (**F**,**G**), and typical permeation assessment model (**H**). The simplistic immersion of epithelial tissue in culture medium (pink) (**A**) is improved by various culture methods accommodating air–liquid interface (ALI) culture conditions (**B**–**E**). The latter included the plasma clot (orange) on watch glass (**B**), agar gel (**C**), raft made using lens paper/rayon acetate floating on medium (**D**), and metal grid (**E**) methods. For epithelial tissue exposure, the chemical substances (green) are placed on the outer surface of the epithelium either via the droplet technique (**F**) or with a cylinder ring (**G**). Permeation assays are usually performed in Franz vertical chambers (**H**), where the permeated substance (yellow) can be measured.

State-of-the-art ex vivo organ culture system development has employed various technical approaches so far, such as the plasma clot or watch glass method, the agar gel method, the raft method, and the grid method [20]. These techniques may be used for the ex vivo culture of epithelial tissues of different origins, such as intestine, upper respiratory tract, or skin, either in full contact with the nutrient medium or in partial contact, i.e., at the air–liquid interface (ALI), as schematically illustrated in Figure 1 [21,22,23]. Previous techniques for exposing tissue to external chemical stimuli have primarily relied on the droplet (Figure 1F) or cylinder ring methods (Figure 1G) [24]. Unfortunately, the droplet approach risks displacement during plate transportation, and its geometry may hinder uniform exposure across the tissue. Meanwhile, the cylinder ring method still carries the risk of movement on the epithelial surface, and the grease residue may affect tissue permeation. The commonly used Franz vertical diffusion chamber system for tissue permeability testing also has limitations, such as a high device-to-tissue volume ratio, requiring large tissue samples (diameter > 6 mm) that could unnecessarily damage and discomfort the donor area while also needing large volumes of culture reagents and space [25]. Therefore, the key challenge is to develop a reliable and reproducible system for exposing oral epithelial tissue to biomaterials and/or drugs through the epithelial barrier, followed by accessible post-analysis for permeation studies.

Based on the above, in the present study, a customized device (Epi-ExPer) was designed and validated to address the unmet need for oral epithelial organ culture while allowing for controlled exposure to exogenous chemical stimuli and facilitating the evaluation of permeation of the tissue after treatments. The Epi-ExPer system was fabricated using a stereolithography (SLA)-based additive manufacturing (AM) method. Human and porcine oral epithelial mucosa tissues were inserted into the device and exposed to resinous monomers commonly released by dental restorative materials. The effect of these xenobiotics on the morphology, viability, permeability, and expression of relevant markers of the oral epithelium was evaluated. The innovative approach presented in this study offers a simple, precise, and reproducible study system of the natural oral mucosa to evaluate interactions with external stimuli, providing a biocompatibility assessment tool with enhanced IVIVE that complies with EU and FDA policies.

## 2. Materials and Methods

A. Epi-ExPer device

### 2.1. Design of the Epi-ExPer Device

The Epi-ExPer device was designed using the computer-aided design (CAD) software AutoCAD 2022 (Autodesk, San Francisco, CA, USA). It consisted of the following two interconnectable components: a lower part and an upper part. The lower part aimed to position the tissue at an ALI state and elevate it, allowing for the collection of permeated substances in the underlying well. The upper part was designed to isolate the desired outer surface of the epithelium, enabling it to be either left untreated or exposed to potentially toxic stimuli under investigation. Sealing rings were also incorporated by bonding them with liquid resin on the cylindrical ridge of the upper Epi-ExPer part, where the tissue would be stabilized, to seal the defined epithelial area at its periphery.

The Epi-ExPer device was designed to precisely fit within the wells of a standard 24-well culture plate, allowing for easy placement of the tissue sample inside a cell incubator during culture, exposure times, and sampling intervals for permeation studies. Additionally, the CAD designs were exported as .stl files and imported into the 3D printing management software PreForm^®^ (version 3.41.0, Somerville, FormLabs, MA, USA), with which mini rafts were generated to provide support for any structurally unsupported components of the design.

### 2.2. Additive Manufacturing and Assembly of the Epi-ExPer Device

The CAD device components were fabricated using the SLA 3D printer Form 3B+ (Formlabs, MA, USA), with a layer thickness of 100 μm. The main parts, including the upper and lower compartments, were manufactured using the biocompatible BioMed Clear resin. The elastic sealing rings were produced with the Elastic Resin 50A (both resins purchased from Formlabs, MA, USA). The printing time for the main parts of 10 devices was approximately 40 min (min), while the sealing rings for 30 devices took around 30 min.

The printed parts were then washed with isopropyl alcohol (IPA) in the FormWash washing chamber (Formlabs, MA, USA), with the main parts washed for 20 min and the sealing rings washed for 10 min. After allowing the parts to fully dry from the IPA, the sealing rings were attached to the edge of the cylinder on the upper parts that contact the tissue. The sealing rings were then chemically bonded to the upper part using BioMed Clear liquid resin, aided by a fine-tipped bonding applicator (Imicryl, Konya, Turkey). The assembled parts were further polymerized in the FormCure polymerization device (Formlabs, MA, USA) for one hour at 60 °C. The supporting raft of the lower parts was removed using a clipper.

The Epi-ExPer devices underwent disinfection by immersing them in 70% ethanol for 15 min, followed by washing with sterile deionized water (3 times × 5 min) and exposure to ultraviolet radiation for 30 min. Thereafter, the devices were stored in 24-well plates until ready for use.

B. Evaluation of toxic stimuli on the Epi-ExPer device

This study was approved (protocol number 62460/2022) by the Ethics and Research Committee of Aristotle University of Thessaloniki (AUTh) and the Ethics Committee of the School of Dentistry, AUTh (protocol number 54/14-03-2022).

### 2.3. Epithelial Organ/Tissue Biopsy and Cultivation

Human normal oral mucosa tissues with non-keratinized/parakeratinized epithelia from the adjacent alveolar mucosa of 3rd molars were obtained from healthy patients during their routine extractions and after patients’ informed consent in the Department of Dentoalveolar Surgery, Implantology and Oral Radiology, School of Dentistry, AUTh. A porcine oral buccal non-keratinized/parakeratinized epithelium was also provided and excised by a local slaughterhouse during the first 2 h after exhaustion. Human or porcine biopsies that were cultivated on the Epi-ExPer devices were obtained using a 5 mm circular biopsy punch (Kai Medical, Solingen, Germany).

The oral epithelial tissues were transferred to the laboratory in ice-cold complete culture medium (CCM) containing 2× antibiotics/antimycotics, where they were aseptically treated in a laminar flow cabinet and cultivated in cell culture incubator (37 °C, 5% CO_2_, relative humidity). The CCM consisted of DMEM: F-12 medium, which was enriched with 10% fetal bovine serum (FBS), 100 U/mL penicillin, 0.1 mg/mL streptomycin, and 0.25 μg/mL amphotericin B (all from PAN Biotech, Aidenbach, Germany).

The cultivated tissues were placed inside the lower part of the Epi-ExPer device. The upper part was then assembled on top of the tissue, and three rubber bands were used to secure it in place, holding the upper part, tissue, and lower part together. Each tissue-loaded Epi-ExPer device was then placed in a 24-well plate. CCM was added to each well surrounding the device so that the tissue was in contact with but not submerged in the CCM. During the exposure-free culturing period, the outer surface of the epithelial tissue was left exposed to air, allowing the tissue to be cultured under ALI conditions.

### 2.4. Exposure of the Epithelial Tissue to Common Toxic Stimuli

Epithelial tissues were placed on Epi-ExPer devices and exposed to commonly released toxic resinous monomers, i.e., (Hydroxyethyl)methacrylate (HEMA) and triethylne glucol dimethacrylate (TEGDMA). The following two monomer concentrations were evaluated: 0.5 mM (TEGDMA-low) and 3 mM (TEGDMA-high) for TEGDMA and 1 mM (HEMA-low) and 4 mM (HEMA-high) for HEMA. Both monomers were first dissolved in ethanol (EtOH) to create a 2 M stock solution. Then, the selected final concentrations were reached by diluting the 2 M solutions at a minimum of 1: 400 to CCM, ensuring that the final ethanol concentration did not exceed 0.25% *v*/*v*. Tissues loaded on the Epi-ExPer devices were exposed to 30 μL of monomer per tissue. The receptor compartment volume of the Epi-ExPer device was 2.4 mL and was filled with either CCM for tissue culture or Hank’s balance salt solution (HBSS) for permeation studies.

### 2.5. Transepithelial Permeation of Calcein

The effect of the resinous monomers (HEMA-low, HEMA-high, TEGDMA-low, and TEGDMA-high) on the permeability of porcine epithelial tissues was evaluated via the transepithelial permeation of calcein. Porcine oral epithelial tissues were mounted on the Epi-ExPer devices (*n* = 3), and the external surface of the epithelial tissues was exposed for 2.5 or 24 h to 30 μL of each of these substances. Tissues exposed only to CCM or 0.25 *v*/*v*% EtOH in CCM acted as blank (CCM) and negative controls (control), respectively. The lower part of the tissue was in contact with CCM. Then, the monomers were replaced by the same amount of calcein solution (1 μg/mL), while the CCM of the lower compartment was replaced by HBSS. Samples of the permeated calcein in the lower compartment were taken at 10-, 20-, 30-, 60-, 90-, 120-, 180-, and 240-min intervals and were analyzed using a spectrofluorometer (RF-5301-PC Fluorescence Spectrophotometer, Shimadzu, Kyoto, Japan). The parameters of steady-state flux (Jss) and apparent permeability coefficient (Papp) were calculated and analyzed. Jss indicated the slope of the plot of the permeated calcein against time, and the Papp was calculated by dividing the Jss by the Cd, where Cd indicated the concentration of calcein of the upper compartment.

### 2.6. Effects of Resinous Monomers on Tissue Viability

The effects of HEMA-low, HEMA-high, TEGDMA-low, and TEGDMA-high on the viability, as expressed by the measurement of the metabolic activity of the porcine epithelial tissue were evaluated via the MTT [3-(4,5-dimethylthiazol-2-yl)-2,5-diphenyltetrazolium bromide] assay. At first, the tissues were weighted, placed on Epi-ExPer devices, and exposed to the monomers for 2.5 or 24 h. Tissues exposed only to 0.25 *v*/*v*% EtOH in CCM acted as the control, while tissues exposed to absolute 100° EtOH acted as positive control (PC). After exposure, the specimens were treated with 5 mg/mL of MTT solution (Life Technologies, San Diego, CA, USA) for 4 h, and then the formed formazan was dissolved in dimethyl sulphoxide (DMSO) for 1 h at 37 °C. Optical density (OD) was measured at 545 nm, with a reference filter of 630 nm against the blank (DMSO) using a microplate reader (Epock, Biotek, Winooski, VT, USA). The percentage (%) OD/tissue weight values compared to the baseline OD of the control were calculated and statistically analyzed (*n* = 4).

### 2.7. Histological Assessment

The effects of the resinous monomers on the morphology and structure of human and porcine epithelial tissues assessed on the Epi-ExPer device were evaluated via hematoxylin and eosin (H&E) staining. Human and porcine tissues were exposed to the relevant monomers for 2.5 or 24 h, respectively. Tissues exposed in 0.25 *v*/*v*% EtOH in CCM acted as control. The tissues were fixed using a 4% paraformaldehyde (PFA) solution for 72 h, gradually dehydrated using serial EtOH dilutions, and embedded in paraffin blocks.

### 2.8. Whole Mount Architecture Evaluation via 3D X-ray Histology

X-ray microfocus computed tomography (μCT)-based 3D X-ray histology (https://xrayhistology.org) [26] was employed to conduct whole-block imaging and study the microstructure of the specimen prior to any physical sectioning. XRH imaging was performed at 80 kVp using a custom Nikon XTH 225 ST system (Nikon Metrology, Castle Donington Derby, UK) at an isotropic voxel size of 6 μm and a geometric magnification of 25×. After the acquisition, the raw radiographic data were reconstructed into 32-bit .vol files using the system’s built-in filtered back projection reconstruction software.

Following reconstruction, a 3D median filter with a 2 × 2 × 2 kernel was applied to the dataset, followed by a 2D unsharp mask with a Gaussian blur factor of 2 pixels. These filtering steps, which were conducted using custom scripts, were essential for enhancing the signal-to-noise ratio, which is crucial for visualization. The pre-processed datasets were then imported into Dragonfly software (6.4, Comet Technologies Canada Inc., Montreal, QC, Canada) for visualization. In Dragonfly, an image moments filter (Kernel size 11) was applied to further denoise the dataset and improve the contrast-to-noise ratio, allowing for clearer visualization and delineation of the tissue from the surrounding wax.

Trained researchers experienced in interpreting XRH data inspected the volumetric histology images using orthogonal cross-sectional views and 3D volume visualizations. Representative cross-sectional views passing through the middle of the exposed area were selected for presentation, as they capture both exposed and unexposed regions. These planes clearly show the epithelium, as well as the underlying lamina propria.

Additionally, maximum intensity and minimum intensity 2.5D (thick-slice modes) images with a slice thickness of 200 μm were used to capture attenuation information across multiple slices. These modes offer a valuable overview of tissue features that is otherwise invisible in single-slice renderings. Thick-slice viewing is a technique that allows the voxel intensity of multiple consecutive slices within a 3D dataset to be rendered onto a single slice based on specific criteria or operations; here, the average intensity and minimum intensity projections are the selected criteria [26]. A thick-slice image represents the result of these criteria or operations.

### 2.9. Histological Sectioning and Imaging

Paraffin blocks, after being scanned with the 3-D X-RAY μCT system, were sectioned with the use of a paraffin microtome (Microm, LabX, Midland, ON, Canada). Obtained 10 μm thick sections were stained with H&E and observed under a light microscope (Nikon Eclipse 80i microscope, Nikon Instruments Inc, Tokyo, Japan).

High-resolution, H&E stained, whole-slide images were captured after scanning the whole section with the Microvisioneer software (Microvisioneer GmbH, Bovenden, Germany), and a Nikon D-eclipse C1 camera (Nikon Instruments Inc., Melville, NY, USA) was used for capturing images at higher magnifications (×10 and ×20).

### 2.10. Immunofluorescence (IF)

The effect of the monomers on the expression of specific markers of human or porcine epithelial tissue was evaluated via IF. Sections of the above-mentioned paraffin-embedded tissues were further assessed. The anti-pCK primary antibody was used for overnight incubation of the specimens at 4 °C (1:50, mouse, IgG monoclonal AE1/AE3, Origene Technologies Inc., Rockville, MD, USA). Then, the following secondary antibody in a buffer of 2% bovine serum albumin in PBS was conjugated to the relevant primary antibody for 1 h at RT: anti-mouse, goat IgG, Ex/Em: 490/525 nm at a 1: 500 dilution (Biotium, Hayward, CA, USA). The tissue was finally observed under a confocal laser scanning microscope (Nikon EZ-C1, CLSM) and further analyzed using the Ez-C1-3.20 software (Nikon Instruments Inc., Amstelveen, The Netherlands).

### 2.11. Statistical Analysis

Statistical analyses were performed using the Prism 8 (GraphPad 8.0, Boston, MA USA) software. The statistical significance was set at *p* < 0.05. Two-way ANOVAs for the factors of time and treatment were performed to analyze the results of calcein permeation and MTT assays, that were followed by Tukey’s post hoc tests. Data were expressed as means ± standard deviation (SD).

## 3. Results

### 3.1. Design of Epi-ExPer Device

**Figure 2:**
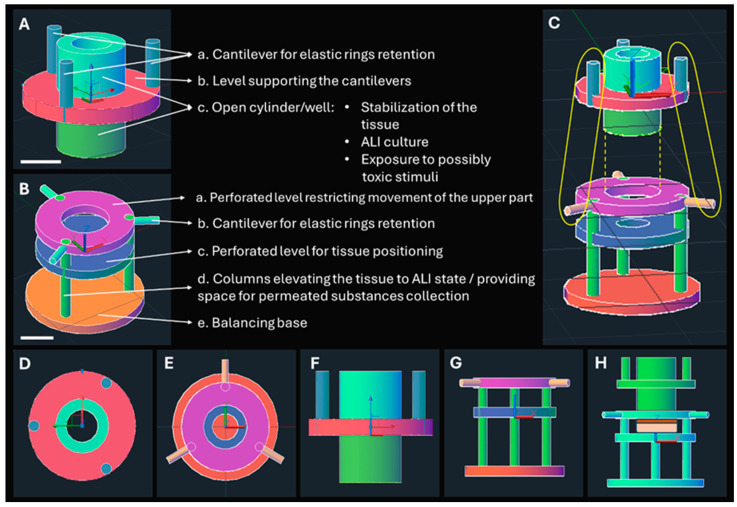
CAD designs of the Epi-ExPer device. The upper (**A**) and lower (**B**) parts, with their compartments ((**A**). a–c and (**B**). a–e) and their roles, are described. Scale bars indicate 3 mm. The relevant position (**C**) of the upper and lower part, where the assembling direction is indicated with a yellow dashed line and the placement of the rubber bands is indicated with the yellow circles. The upper view of the upper (**D**) and lower (**E**) parts and the side views (**F**,**G**) are depicted. The assembled device with an animated epithelial tissue is illustrated (**H**).

The CAD schemes of the Epi-ExPer device, which was designed to precisely fit into a 24-well plate, are illustrated in Figure 2. The upper part (Figure 2A) features an open cylinder with an inner diameter of 3 mm, which provides a free surface area of 28.3 mm^2^ for ALI culture or exposure of the multilayered epithelium to tested substances in solution. The outer diameter of the cylinder is 4.5 mm, resulting in a 1.5 mm width between the inner and outer surfaces. In this area, an elastic sealing ring is attached to stabilize the tissue (c in Figure 2A).

The lower part was designed with a 3 mm-diameter free area (c in Figure 2B), allowing the tissue to receive nutrients from the culture medium (CCM) that fills the culture well up to the tissue level. This enabled the permeated substances to perfuse through the entire tissue and be collected in the underneath area for further quantification. Additionally, the upper part was designed with an inner diameter of 5 mm (a in Figure 2B), allowing the open cylinder to glide inside during the assembly of the tissue and device (Figure 2H).

### 3.2. Manufactured and Assembled Epi-ExPer Device

**Figure 3:**
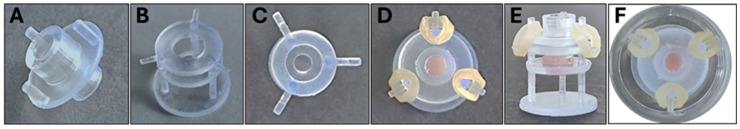
Photographs of the additively manufactured via stereolithography Epi-ExPer device. Upper (**A**) and lower (**B**,**C**) parts and the assembled device loaded with epithelial tissue (**D**,**E**) inside a 24-well plate (**F**).

The manufactured parts of the Epi-ExPer device are shown in Figure 3A–C and then shown as assembled with the tissue and placed in the 24-well plate (Figure 3D–F). The receptor compartment, which is the space under the lower part that restricts the movement of the upper part, can accommodate a 2.4 mL volume of liquid. Similarly, 2.4 mL of HBSS is expected to be added to the 24-well plate surrounding the Epi-ExPer device to measure the permeation of substances under study. The open well of the upper part can hold up to 30 μL of liquid.

### 3.3. Transepithelial Permeation of Calcein Assay

**Figure 4:**
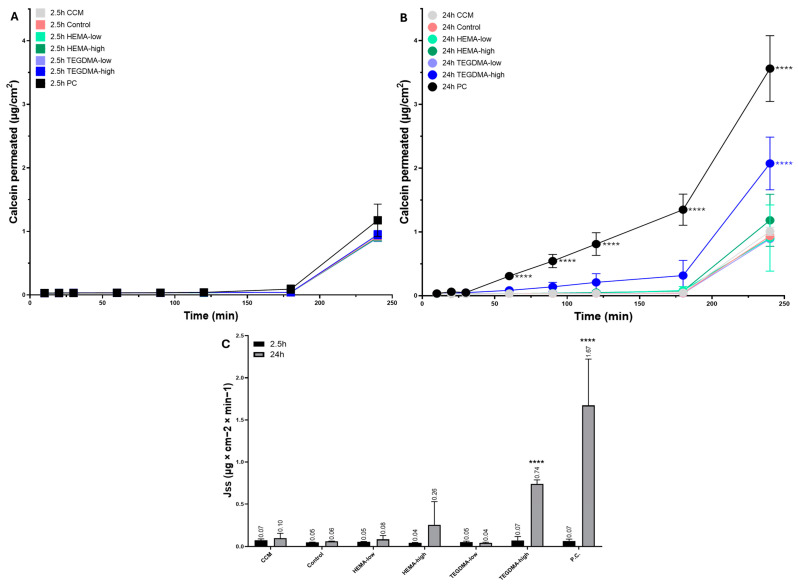
Permeation of calcein on porcine tissue loaded on Epi-ExPer device after 2.5 and 24 h of exposure to HEMA-low, HEMA-high, TEGDMA-low, and TEGDMA-high. Treatment with the resinous monomers under study after 2.5 h (**A**) and 24 h (**B**) and the Jss (**C**) for both time points are depicted. The CCM group was exposed only to the culture medium, and the control group was exposed to 0.025% EtOH/CCM, which is the diluent of HEMA and TEGDMA, while the positive control (PC) group was treated with 100° EtOH. Asterisks indicate statistically significant differences between the tested materials compared to the relevant control (**** *p* ≤ 0.0001).

The permeation of calcein results for the different treatments of the porcine epithelial tissues on the Epi-ExPer device is shown in Figure 4. During the 2.5 h of treatment, no statistically significant differences in the amount of the permeated calcein or the relevant Jss and Papp parameters were observed among the groups (*p* > 0.05). During the 24 h exposure period, the permeation of calcein was increased between the PC and all the other groups at the 90 min measurement and continuing thereafter (all *p* < 0.0001). Additionally, the TEGDMA-high group exhibited significantly elevated calcein permeation at the 240 min timepoint compared to every other group (*p* < 0.0001). The effect of TEGDMA-high on the permeability of the porcine epithelial tissue was confirmed by the parameters of Jss and Papp, which were statistically significant compared to the control (*p* < 0.0001).

The parameters Jss and Papp for the permeation of calcein are presented in Table 1.

### 3.4. Effect of Resinous Monomers on Tissue Viability

**Figure 5:**
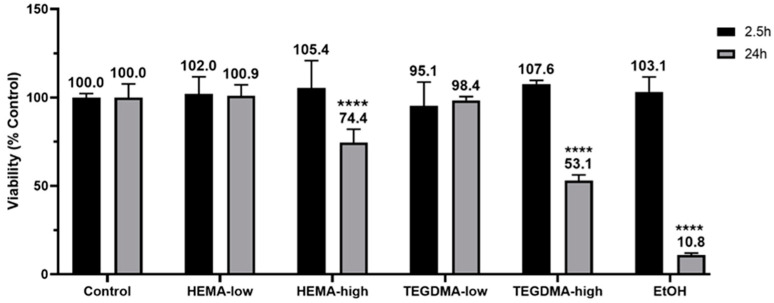
Tissue viability (*n* = 3) after exposure to HEMA and TEGDMA for 2.5 h (black columns) and 24 h (gray columns). Mean values are annotated above each column, and asterisks indicate statistically significant differences between the tested materials compared to the relevant control (**** *p* ≤ 0.0001).

The viability of the epithelial tissues (Figure 5) was affected by the tested resinous monomers in a concentration- and time-dependent manner. After 24 h of treatment, HEMA-high reduced tissue viability to 74.4 ± 7.6%, while TEGDMA-high decreased it to 53.1 ± 3.1% (both *p* ≤ 0.0001). The positive control, which was treated with absolute ethanol, exhibited a drastic reduction of tissue viability down to 10.8 ± 1.1% (*p* ≤ 0.0001). However, no statistically significant changes in viability were observed during the 2.5 h treatment period (*p* > 0.05).

### 3.5. Whole Mount Architecture Evaluation via 3D X-ray Histology

**Figure 6:**
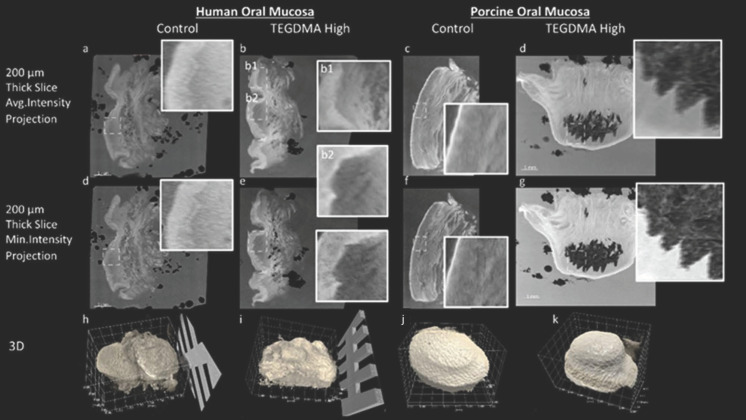
Three-dimensional X-ray histology imaging of human and porcine oral mucosa. Comparison of control and high-concentration TEGDMA-treated tissues for both human and porcine oral mucosa samples. The top and middle rows (**a**–**g**) show 200 μm thick-slice projections for average intensity (top row) and minimum intensity (middle row), highlighting tissue microstructure. Insets provide zoomed-in views of the dashed line-outlined areas. Insets (**b1**,**b2**) provide zoomed-in views of the exposed (**b2**) and adjacent unexposed (“in situ control”, (**b1**)) areas of the human TEGDMA-high sample shown in (**b**). Three-dimensional reconstructions (**h**–**k**) illustrate the whole tissue, delineated from the wax before any physical sectioning, in which the indentation of the cylindrical well is clearly visible, outlining the exposed epithelium area in the center.

XRH results are presented in Figure 6 for both the human and porcine tissue experiments, comparing the control and the most damaged sample exposed to high-concentration TEGDMA treatments. The 3D micrographs (Figure 6—bottom row) illustrate the tissue delineated from the wax in relation to the histology cassette prior to any physical sectioning. In all images, the indentation of the cylindrical well is clearly visible, outlining the exposed epithelium area in the center, with the unexposed tissue surrounding this confined space.

A comparison between the control and TEGDMA-high treatments for both human and porcine specimens indicates the influence of the chemical compounds on the epithelium; also see Supplementary Video S1. Additionally, the underlying deeply located connective tissue shows areas of lower attenuation, which result from the presence of loosely packed collagenous/fibrous tissue components and adipose tissue. These darker, lower-density areas are particularly apparent in the 200 μm average intensity projection and the minimum intensity projections across all TEGDMA-high samples.

A closer examination of the zoomed-in inserts (outlined with dashed lines) reveals that this looser organization of stromal tissues is more apparent in lower attenuation areas (darker regions in the areas exposed to the resinous monomers (Figure 6(b2))) compared to the control (Figure 6a) and in situ control samples (Figure 6(b1)), depending on the site of the biopsy.

**Figure 7:**
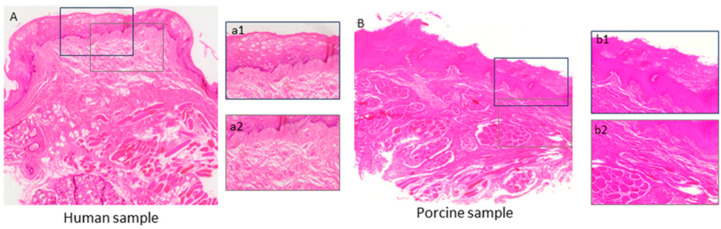
Whole-section histological scanning images of human (**A**) and porcine (**B**) oral mucosa specimens exposed to high concentrations of resinous monomers, which were previously scanned with the 3D X-ray μCT (Figure 6). The human mucosa exhibited severely altered cell morphology and cell damage due to extensive vacuolation in the upper two-thirds of the epithelium (**a1**). Similar, though less pronounced (covering only half of the epithelium), lesions were observed in the porcine epithelium (**b1**). In contrast, the underlying lamina propria was not substantially affected in either the human or porcine mucosa (**a2**,**b2**, respectively), and there were no significant alterations in collagen band density or tissue disruption.

Whole-section histological imaging was performed to further elucidate the XRH findings and the nature of the lower attenuation areas. This microscopic evaluation assessed the effect of the resinous monomers on the tissue integrity of the previously 3D X-ray μCT scanned oral mucosa specimens of both human and porcine origin. The scanning revealed that resinous monomers evoked severe disorganization and cell damage in the epithelial layer while having no effect on the underlying lamina propria (Figure 7). The collagen fibers in the lamina propria maintained their normal orientation, architecture, and density. Furthermore, a well-organized area of connective tissue was observed in close proximity to the basal epithelial membrane of all samples, excluding the possibility of connective tissue destruction by TEGDMA and HEMA. The areas of loose organization in the deeper layer of the lamina propria are a result of the tissue’s physiological architecture, which is further accentuated by surgical handling and experimental procedures. These areas became more obvious in the exposed samples, most likely due to the disorganization of the epithelial layer, which made them frailer during handling.

### 3.6. Histological and IF Analyses of Exposed Human Epithelial Tissues

**Figure 8:**
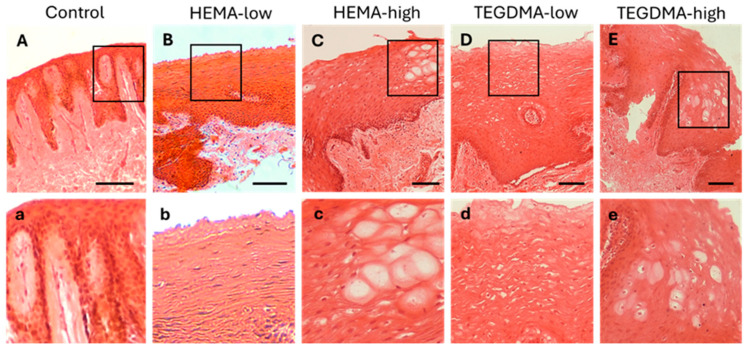
H&E photomicrographs of 10 μm human oral mucosa sections treated with 0.25% *v*/*v* EtOH in CCM (**A**,**a**), HEMA-low (**B**,**b**), HEMA-high (**C**,**c**), TEGDMA-low (**D**,**d**), or TEGMA-high (**E**,**e**). Treatment with HEMA and TEGDMA for 2.5 h resulted in severe disorganization of normal tissue architecture (**C**–**E**) with altered cellular morphology of epithelial cells (**c**–**e**). Vacuolar degeneration of epithelial cells was observed at high HEMA and TEGDMA concentrations (**c**,**e**) pointing to their dose-dependent effect, as higher concentrations of both resins resulted in more severe cellular and tissue damage. Scale bar = 100 μm.

H&E staining of human epithelium (Figure 8) revealed that treatment with HEMA led to mild disorganization of the superficial epithelial layer, as well as focal intraepithelial edema and epithelial vacuolation in the lower epithelial layers. In contrast, treatment with TEGDMA, particularly at higher concentrations, resulted in a more diffuse disruption of the upper epithelial layer and extended vacuolation and degeneration of epithelial cells in the lower epithelial layers compared to the control.

**Figure 9:**
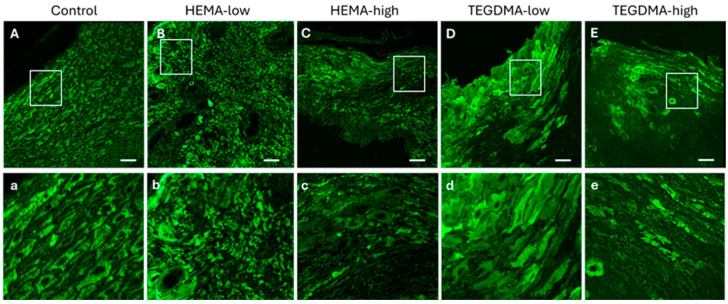
CLSM photomicrographs of 10 μm human oral mucosa sections stained against the anti-pCK. Oral mucosa specimens placed in the Epi-ExPer device were either treated with 0.25% *v*/*v* EtOH in CCM (**A**,**a**), HEMA-low (**B**,**b**), HEMA-high (**C**,**c**), TEGDMA-low (**D**,**d**), or TEGMA-high (**E**,**e**). Treatment with HEMA and TEGDMA for 2.5 h, resulted in epithelial atrophy (**C**,**c**,**E**,**e**), altered morphology (**D**,**d**), vacuolar degeneration of epithelial cells (**C**,**c**), and severe de-organization of the normal tissue architecture. HEMA and TEGDMA’s effect on the integrity of oral epithelia was dose-dependent, as higher concentrations of both resinous monomers resulted in more severe cellular and tissue damage. Scale bar = 100 μm.

The observed effects on epithelial tissue were more evident with IF after staining with the pCK marker and visualization using CLSM (Figure 9). Specifically, the dose-dependent corrosive impact of HEMA and the even more pronounced effect of TEGDMA, causing severe disruption of epithelial architecture and induction cellular vacuolation, were clearly evident compared to normal human epithelial tissues.

### 3.7. Histological and IF Analyses of Exposed Porcine Epithelial Tissues

**Figure 10:**
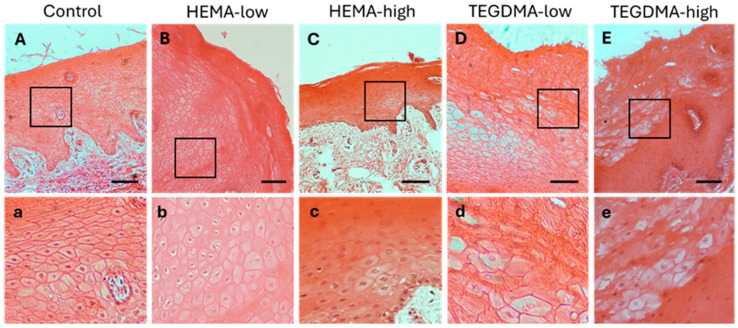
H&E photomicrographs of 10 μm porcine oral mucosa sections treated with 0.25% *v*/*v* EtOH in CCM (**A**,**a**), HEMA-low (**B**,**b**), HEMA-high (**C**,**c**), TEGDMA-low (**D**,**d**), or TEGMA-high (**E**,**e**). Treatment with HEMA (**B**,**C**) and TEGDMA (**D**,**E**) for 24 h resulted in severe disturbance of epithelial architecture, altered cellular morphology in TEGDMA-treated mucosa (**d**,**e**), and vacuolation of epithelial cells ((**b**–**d**,**f**) for HEMA and TEGDMA, respectively), even at deeper epithelial layers. TEGDMA posed a more severe effect on epithelial integrity, even at low concentrations (**D**,**E**). Scale bar = 100 μm.

**Figure 11:**
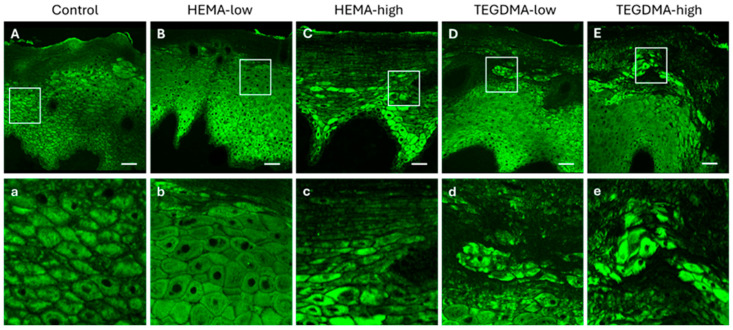
CLSM photomicrographs of 10 μm porcine buccal mucosa sections stained against anti-pCK. Oral mucosa specimens were either treated with 0.25% *v*/*v* EtOH in CCM (**A**,**a**), HEMA-low (**B**,**b**), HEMA-high (**C**,**c**), TEGDMA-low (**D**,**d**), or TEGMA-high (**E**,**e**). Treatment with HEMA and TEGDMA for 24 h resulted in epithelial atrophy (**C**–**E**,**c**–**e**), altered morphology of epithelial cells, and severe disturbance of epithelial architecture. TEGDMA induced a more severe effect on epithelial integrity, even at low concentrations (**D**,**d**). HEMA and TEGDMA’s effect on the integrity of oral epithelium was dose-dependent, as higher concentrations of both resins resulted in more severe cellular and tissue damage. Scale bar = 100 μm.

When porcine buccal mucosa with a non-keratinized/parakeratinized epithelium was treated with HEMA at low and higher concentrations, a diffuse vacuolation of epithelial cells in several layers was observed (Figure 10 and Figure 11). However, in contrast to the human epithelium, no disorganization of the upper epithelial layer was noticed compared to the non-exposed controls. Conversely, the influence of both lower and higher concentrations of TEGDMA in porcine buccal mucosa led to extended disorganization of the upper epithelial layer and vacuolation of the underlying epithelial cell layers.

## 4. Discussion

The ongoing advancement of novel dental materials that interact with the human oral mucosa, along with the emergence of new trans-mucosal drug delivery systems, necessitates rigorous evaluation of these new agents [27,28,29]. Assessing the impact on the structural integrity, viability, and permeability of oral mucosa is critical, and therefore the development of reliable assessment tools is essential. Engineered oral mucosal equivalents have been developed for clinical use, as well as for in vitro studies of biocompatibility, mucosal irritation, disease, and other fundamental oral biological processes [11,12,30,31]. In addition, multiple studies have described the successful construction of full-thickness engineered human oral mucosa through the cultivation of oral keratinocytes with or without fibroblasts on collagen substrates [12,13,14,15,16], as well as dynamic oral mucosa cultures on-a-chip [17]. Although significant advancements have been made, these engineered systems still fall short of accurately mimicking the complex barrier functions and microenvironment of the native oral epithelium. Yet, this level of biomimicry is often crucial for the spatiotemporal evaluation of host–material interactions and responses that reflect both healthy and diseased states.

This study introduces the Epi-ExPer device, which was developed to address certain limitations of tissue engineering models and two-dimensional cell cultures as alternatives to in vivo animal testing. The Epi-ExPer device aims to provide a reliable platform for the preclinical evaluation of new dental materials and oral care products, investigation of oral pathologies, and study of fundamental biochemical and biophysical processes in oral tissues.

The Epi-ExPer device was designed to provide a reliable, easy-to-use, and practical tool for ex vivo oral mucotoxicity studies. The first goal was to integrate multiple functionalities, including tissue culture, exposure, and permeability evaluation, into a single device. Once the epithelial tissue is assembled, no additional equipment is needed for exposure (e.g., clone ring), and the tissue does not require transfer to another device for subsequent permeability evaluation (e.g., Franz chambers). Another aim was to enable a more sustainable, high-throughput approach for ex vivo testing compared to existing models by designing the device to fit in standard 24-well culture plates [32]. This reduces the required tissue area and reagent volume while increasing the experimental data-to-resources ratio.

The device was designed to maintain the natural orientation of the epithelial tissue during the culture stage. The lower part of the device accommodates an ALI potential, where the epithelium is in contact with the air and the underlying lamina propria is immersed in the culture medium [33,34,35]. This setup closely mimics the clinical conditions, as the agents to be evaluated, such as dental materials, microbial agents, or drugs, come into contact only with the epithelial side of the tissue [36].

The Epi-ExPer device incorporates a compartmentalization feature inspired by a modified skin culture system suggested by Companjen et al. [37]. The authors perforated the filter of a culture insert and placed the dermis of the tissue through the hole, while the epidermis was positioned higher than the filter level, exposing only that area to the factors under study. Similarly, the Epi-ExPer device has an upper well that remains empty during the ALI culture of the tissue but is filled with the agents to be evaluated during the exposure experiments. This upper well provides controlled exposure to exogenous factors, without the risks associated with other techniques, such as the movement inherent in droplet exposure or the potential for sealing material residues that can occur with the clone ring technique [24].

Although the Franz vertical diffusion chambers are considered the gold standard for ex vivo permeation testing [38,39], the literature has highlighted several shortcomings, including the relatively large volumes of reagents they require, their bulky size, and their fragility [40]. Many variations of the Franz chamber have been introduced, but their scopes and intended usages differ from the Epi-ExPer device developed in this study. For example, Sil et al. fabricated non-fragile, cost-effective Franz chamber analogs using SLA AM [25], while Munt et al. developed a diffusion chamber targeting the examination of topical spray formulations [40]. Neil et al. used vertical Franz diffusion chambers as culture platforms and demonstrated the successful long-term culture of human skin beyond 9 days [41]. In contrast, the Epi-ExPer device was developed using a bottom-up design approach specifically aimed at optimizing the culture, exposure, and permeation evaluation of human oral mucosa.

The Epi-ExPer device was used to assess the biocompatibility of dental materials. For this purpose, HEMA and TEGDMA were selected, as they are the most released xenobiotics from resin-based dental restorative materials that come into direct or indirect contact with oral mucosa [42,43]. These monomers have been extensively studied for their biological effects in vitro [44,45,46]. The time points of 2.5 and 24 h were chosen based on pharmacokinetic studies, which have shown that after the in vivo oral application of resinous monomers, the highest concentrations are detected during the first few hours, while they are almost eliminated by 24 h [42,47]. To the best of the authors’ knowledge, there is no previous study that has evaluated the effects of these two dental resinous monomers, HEMA and TEGDMA, on ex vivo oral mucosa.

In the present study, porcine oral buccal samples were used alongside human biopsies to evaluate histological alterations and the pCK expression of the oral mucosa, as porcine tissue has been found to most closely resemble human oral mucosa in terms of permeability [48]. The porcine mucosa has a greater number of epithelial layers compared to that of humans [49]. Consequently, the human oral mucosa tissue was exposed for 2.5 h, while the porcine oral mucosa was exposed for 24 h. The increased thickness of the porcine oral epithelium may contribute to the resilience of the tissue to the evaluated resinous monomers. The study findings indicate that the impact of both monomers was dose-dependent, with higher concentrations resulting in more pronounced changes. Additionally, TEGDMA appeared to cause more severe lesions compared to HEMA, although both monomers induced changes in the mucosae. A combination of 3D X-ray histology, followed by whole-section scanning of high-resolution, H&E stained, whole-slide images, revealed that the observed lesions were restricted into the epithelium without causing any adverse effects or histological changes in the architecture of the underlying lamina propria, other than those related to surgical handling and experimental post-analysis. High-throughput 3D X-ray histology can be a very useful and well-established method to be used in cases seeking to locate the lesions in a complex whole-mount sample containing both exposed (tissue area delimited within the exposure ring of the Epi-ExPer device) and unexposed (in situ control, i.e., area on the outer surface of the Epi-ExPer ring) areas (Figure 6 and Figure 7), providing valuable information for the subsequent histological sectioning and analysis [26].

For the toxicity evaluation, the MTT assay was performed, which is one of the most commonly used assays to determine cell viability for both in vitro and ex vivo testing [50,51]. According to the Organisation for Economic Co-operation and Development (OECD) Guidelines for the Testing of Chemicals (TG431 and 439), the MTT assay is an essential tool for establishing a reconstructed human epidermis model [29,52]. In a previous study by Wang et al., the researchers conducted the MTT assay, along with permeability testing and histological analysis, to determine the most suitable ex vivo animal model for mimicking human mucosa, supporting the use of porcine models [53]. In the present study, the MTT assay was conducted on porcine mucosa after 2.5 and 24 h of exposure to assess the toxic effects of HEMA and TEGDMA at different concentrations. Negative and positive controls were tested in accordance with the OECD Guidelines. The results showed that the highest concentrations of HEMA and TEGDMA had the greatest negative impact on tissue viability, which was expected based on relevant in vitro cell culture studies reported in the literature [43,54].

Permeability assays are commonly conducted in drug delivery studies, as they provide insights into the continuity of the epithelium, which can serve as an indirect assessment of the impact of a toxic substance. The Franz diffusion cell is considered the state-of-the-art permeability test, as it is a static device consisting of a donor and a receiver chamber, allowing for the evaluation of a specific substance’s permeation through tissue in both in vitro and ex vivo models [48,55]. Most studies in the literature employ the Franz cell as their primary permeability assay [56,57,58]. For example, Elefteriadis et al. used a buccal bovine model in Franz diffusion cells to assess the performance of a mucoadhesive buccal film for the simultaneous delivery of lidocaine and ketoprofen [59]. Similarly, Farias et al. utilized the Franz cell setup to study the permeation of a composite polymer-based lyophilized wafer through multiple models, i.e, a reconstructed human epidermis (RhE) commercially available model, a porcine buccal tissue, and an artificial buccal membrane [60], while some studies have also employed the Ussing chamber [61,62]. However, the design of the Franz cells allows for the testing of a specific diameter of tissue of at least 6 mm or more. The Epi-ExPer device presented in the present study allows for the use of smaller tissue samples, even as small as 5 mm in diameter, thereby conserving animal or human tissues.

## 5. Conclusions

The developed Epi-ExPer device represents a promising tool for the ex vivo assessment of external stimuli and/or xenobiotics that come into contact with the oral mucosa. The design and manufacturing of the device facilitate the oriented culture of the oral mucosa tissue under ALI conditions, the delimited exposure to external chemical agents and/or biomaterials, and the reproducible evaluation of tissue permeability immediately post-treatment. Furthermore, the device accommodates a cost-effective, multi-use, and high-throughput approach for the evaluation of newly developed biomaterials and pharmaceutical agents interacting with the oral mucosa. A proof-of-principle analysis of the Epi-ExPer system in the present study also suggested a dose-dependent corrosive effect of resinous monomers (HEMA and TEGDMA) commonly released by resin-based dental restorative materials, which is, however, restricted, even at high concentrations, within the epithelium, leaving the underlying lamina propria unaffected, even after 24 h of exposure. Further studies employing dental materials in direct contact with the proposed ex vivo oral mucosa assessment system in comparison with animal studies (e.g., direct contact of the materials with animal buccal mucosa) will provide more insights into the validity and IVIVE potential of the proposed biocompatibility assessment tool.

## 6. Patents

The Epi-ExPer device is under evaluation for being patented.

## Figures and Tables

**Table 1:** Steady-state flux (Jss) and the apparent permeability coefficient (Papp) for the calcein permeation through porcine oral buccal mucosa loaded on the Epi-ExPer device after 2.5 and 24 h of exposure to two concentrations of the resinous monomers HEMA and TEGDMA.

Group	J_ss_ · 10^−4^ ± SD (μg × cm^−2^ × min^−1^)	P_app_ · 10^−6^ ± SD (cm × h^−1^)
CCM—2.5 h	0.72 ± 0.18	1.97 ± 0.14
Control—2.5 h	0.49 ± 0.04	2.90 ± 0.71
HEMA-low—2.5 h	0.55 ± 0.05	2.19 ± 0.21
HEMA-high—2.5 h	0.44 ± 0.05	1.76 ± 0.20
TEGDMA-low—2.5 h	0.53 ± 0.10	2.11 ± 0.42
TEGDMA-high—2.5 h	0.72 ± 0.46	2.87 ± 1.85
EtOH—2.5 h	0.66 ± 0.21	2.66 ± 0.84
CCM—24 h	0.98 ± 0.58	3.94 ± 2.31
Control—24 h	0.61 ± 0.04	2.46 ± 0.14
HEMA-low—24 h	0.85 ± 0.45	3.40 ± 1.80
HEMA-high—24 h	2.56 ± 2.73	10.25 ± 10.92
TEGDMA-low—24 h	0.44 ± 0.07	1.77 ± 0.27
TEGDMA-high—24 h	7.38 ± 0.50	29.51 ± 1.98
EtOH—24 h	16.73 ± 5.50	66.91 ± 21.98

## Data Availability

Data can be made available upon request.

## Supplementary Materials

Video S1: A 10× slice minimum projection roll (left) and a 10× slice average projection roll (right), highlighting a lower-density region within the epithelial area in human TEGDMA-high samples, as indicated by arrows and insert. This region is associated with altered cellular morphology and epithelial cell vacuolation, as in Figure 8. Video available at https://safesend.soton.ac.uk/pickup?claimID=XuU847S67oPH9x4g&claimPasscode=6NkC9hfjRtTvWZ9h (accessed on 14 September 2024), Claim ID: XuU847S67oPH9x4g, Claim Passcode: 6NkC9hfjRtTvWZ9h.
